# Analysis of factors affecting acute fatigue in patients with acute ischemic stroke: A single-center study from China

**DOI:** 10.1016/j.clinsp.2025.100667

**Published:** 2025-05-02

**Authors:** Yunkun Lin, Wenjing He, Junwei He, Limei Liu

**Affiliations:** a Affiliated Hospital of Putian University, Putian, China; b Meizhouwan Vocational Technology College, Putian, China

**Keywords:** Poststroke fatigue (PSF), Fear of Disease Progression, Social Support, Influencing Factors

## Abstract

**Objective:**

Examination of the current status of acute fatigue in patients with acute ischemic stroke and analysis of contributing factors.

**Methods:**

A convenience sampling method was utilized to recruit 420 patients diagnosed with acute ischemic stroke, who were admitted to the affiliated hospital of Putian University in Fujian Province, China, between October 2022 and December 2023. Data collection involved the administration of a general information questionnaire, the Fatigue Severity Scale (FSS), the Fear of Progression Questionnaire (FoP-Q-SF), the Perceived Social Support Scale (PSSS), the Barthel Index, and the Modified Rankin Scale (MRS).

**Results:**

The mean score for Post-stroke Fatigue (PSF) was 3.08 ± 1.52, and the prevalence rate of elevated PSF was 37.4 %. Unconditional logistic regression analysis revealed that social support functioned as a protective factor against fatigue in patients with acute ischemic stroke. Conversely, being female, having chronic comorbidities, having a stroke located in the basal ganglion, high Modified Rankin Scale (MRS) scores, poor self-care ability, and fear of disease progression were identified as risk factors for fatigue (*p* < 0.05 or *p* < 0.01).

**Conclusion:**

Perceived Stress Factor (PSF) is a common and serious consequence following stroke, which considerably impacts patients’ recovery and overall quality of life. Consequently, clinical practice should prioritize the identification and management of PSF through early intervention and targeted therapeutic strategies to enhance patient prognosis and quality of life. Additionally, further research is warranted to investigate the underlying biological mechanisms of PSF and to develop potential intervention strategies. This study adopted a cross-sectional research design.

## Highlights

• This study aimed to analyze factors affecting acute fatigue in acute ischemic stroke patients. A single-center convenience sample of 420 patients was used.
• Female gender, chronic comorbidities, etc. were identified as risk factors for acute fatigue, while social support was a protective factor, guiding clinical interventions.
• There was a positive correlation between fear of disease progression and acute fatigue. Alleviating fear may improve patients' fatigue and quality of life.
• The incidence of acute fatigue in acute ischemic stroke patients was 37.4 %, which affected rehabilitation. It's crucial to intervene for better rehabilitation outcomes.

## Introduction

Stroke is one of the leading causes of death and disability worldwide.^1^^,^^2^ Despite advancements in acute stroke treatment that have increased survival rates, many survivors experience long-term health issues post-stroke, with Post-stroke Fatigue (PSF) being one of the most prevalent and consequential complications.^3^^,^^4^ PSF not only severely affects the functional status and rehabilitation process of patients but also significantly impairs their quality of life.^5^, ^6^, ^7^ However, despite the growing clinical focus on PSF, its precise pathophysiology and risk factors remain not fully elucidated.^8^ This cross-sectional study is designed to further explore the influencing factors of acute fatigue in patients with acute ischemic stroke, aiming to provide more targeted evidence for clinical management. Currently, although there are some studies on this topic, there are still limitations in sample selection and research methods in many of them, which may affect the accuracy and universality of the results.

## Methods

### Patients

This study used a convenience sampling method to select 420 patients with acute ischemic stroke who were hospitalized in the neurology department of the Affiliated Hospital of Putian University in Fujian Province, China, between October 2022 and December 2023. Among these patients, 252 (60.0 %) were male and 168 (40.0 %) were female, with ages ranging from 24 to 80 years and an average age of 62.72±10.25 years.

Inclusion Criteria: 1) Diagnosis meets the criteria established by the “Chinese Guidelines for the Diagnosis and Treatment of Acute Ischemic Stroke 2023″, confirmed by MRI or CT imaging. 2) Age between 18 and 80 years. 3) No cognitive impairment, stable condition, and able to complete scale assessments. Cognitive impairment was evaluated using the Mini-Mental State Examination (MMSE), and patients with a score above 24 were considered to have no cognitive impairment. The stability of the patient's condition was determined by continuous monitoring of vital signs and neurological symptoms for at least 24 hours without significant changes. The ability to complete scale assessments was evaluated through a pre-assessment interview to ensure that patients could understand and respond to the questions. 4) Informed consent was obtained and voluntary participation in the study.

Exclusion Criteria: 1) Modified Rankin Scale (MRS) score > 4, a score above 4 on the MRS indicates severe neurological deficits and significant disability, which may interfere with the accurate assessment of fatigue symptoms and the representativeness of the study sample for general acute ischemic stroke patients. 2) History of psychiatric disorders. Psychiatric disorders may confound the perception of patients and reporting of fatigue, thus affecting the reliability of the research results. 3) Presence of cancer or significant organ failure.

It should be noted that the convenience sampling method used in this study may introduce selection bias, and the sample may not be fully representative of all acute ischemic stroke patients. Future research could consider using more random and comprehensive sampling methods to improve the generalizability of the findings.

### Research tools

1) General survey form: This includes data on factors such as gender, age, marital status, family income, living arrangements, body mass index, familial medical history, concurrent chronic conditions, stroke site, and the recurrence of stroke incidents.
2) Fear of Progress Questionnaire-Short Form (FoP-Q-SF): Including two dimensions (physical and family/social) with 12 items, higher scores signify a greater fear of disease progression. The total score ranges from 12 to 60, with scores above 34 indicating dysfunctional fear. The Cronbach’s alpha coefficient for this scale is 0.883.
3) Fatigue Severity Scale (FSS): Assessing fatigue in the acute phase of acute ischemic stroke. Higher scores denote a more severe level of fatigue. A mean score above 4 is considered stroke-induced fatigue. This is a unidimensional scale with nine items, of which the total score is the mean. The Cronbach’s alpha coefficient for this scale is 0.928.
4) Perceived Social Support Scale (PSSS): With two dimensions (intra-family and extra-family) and 12 items. Higher scores indicate a higher perceived level of social support. Support status is classified as low (12‒36), moderate (37‒60), and high (61‒84) based on the total score. The Cronbach’s alpha coefficient for this scale is 0.899.
5) Barthel Index: Utilized to evaluate patients’ activities of daily living. Higher scores denote better self-care abilities. Scores are categorized as follows: full independence at 100, mild dependence is 61‒99, moderate dependence is 41‒60, and severe dependence is ≤ 40.
6) Modified Rankin Scale (MRS): A standard instrument for clinicians to assess the extent of neurological impairment. Higher scores signify a greater degree of disability.

### Survey methodology

Within 48 – 72 hours after patient admission, once the patient’s condition has stabilized, the researchers will present the significance, objectives, and confidentiality principles of the study to the patient. Following the acquisition of informed consent, the researchers will proceed with the questionnaire survey. For patients who are able to complete the questionnaire independently, researchers will provide guidance on how to fill it out based on their specific circumstances and promptly address any questions. For those unable to complete the questionnaire on their own, researchers will orally present the questionnaire items and record responses as provided by the patients. All questionnaires were distributed and collected on-site, with each one individually checked to ensure its completeness.

Our study was approved by the ethics committee of the Affiliated Hospital of Putian University (approval n° 2023,074). Finally, a total of 450 questionnaires were distributed for this survey, with 420 valid responses collected, yielding an effective response rate of 93.3 %.

### Statistical analysis

Statistical analyses were performed using SPSS version 23.0. Continuous data are presented as mean ± standard ($\bar{x}$±s) deviation and were analyzed with the *t*-test. Categorical data are expressed as percentages and were evaluated using the Chi-Square (χ^2^) test. Correlation analysis was conducted using scatter plots generated with GraphPad Prism version 8.0.1. Furthermore, unconditioned logistic regression and other statistical methods were utilized. Statistical significance was defined as p-values < 0.05 or < 0.01.

## Result

### FSS scoring of PSF in patients

The results indicated that the mean score for PSF among patients with acute ischemic stroke was (3.08 ± 1.52), and 157 patients (37.4 %) experienced PSF (Table 1).

**Table 1:** FSS scoring of PSF Symptoms in patients with acute ischemic stroke (*n* = 420, $\bar{x}$ ± *s*).

Number	Item	Score
1	My motivation is lower when I am fatigued.	4.14±1.93
2	Exercise brings on my fatigue	3.65±1.82
3	I am easily fatigued.	3.34±1.73
4	Fatigue interferes with my physical functioning	2.83±1.62
5	Fatigue causes frequent problems for me	2.68±1.55
6	My fatigue prevents sustained physical functioning	2.68±1.49
7	Fatigue interferes with carrying out certain duties and responsibilities	2.45±1.43
8	Fatigue is among my most disabling symptoms	2.21±1.12
9	Fatigue interferes with my work, family, or social life	2.11±1.05

### Univariate analysis

The results revealed statistically significant differences in PSF among patients with acute ischemic stroke based on variables such as Sex, household income, cohabitation status, family history, presence of chronic diseases, stroke location, self-care ability, MRS score, and perceived social support (*p* < 0.05 or *p* < 0.01), as detailed in Table 2.

**Table 2:** Univariate analysis of PSF Incidence in acute ischemic stroke patients based on demographic data (*n* = 420), n ( %).

Item	Case number	Non-fatigue group (*n* = 263)	Fatigue group (*n* = 157)	χ^2^	p
Sex					
Male	252	200 (79.4)	52 (20.6)	75.476	<0.01
Female	168	63 (37.5)	105 (62.5)		
Age(years)					
24∼44	25	12 (48.0)	13 (52.0)	2.724	>0.05
45∼59	117	72 (61.5)	45 (38.5)		
60∼74	235	151 (64.3)	84 (35.7)		
≥75	43	28 (65.1)	15 (34.9)		
Marital status					
Married	364	230 (63.2)	134 (36.8)	0.605	>0.05
Single	14	9 (64.3)	5 (35.7)		
Divorced or widowed	42	24 (57.1)	5 (35.7)		
Monthly household income (Yuan)					
< 2000	103	45 (43.7)	58 (56.3)	24.369	<0.01
2000∼4999	160	102 (63.7)	58 (36.3)		
≥ 5000	157	116 (73.9)	41 (26.1)		
Cohabitation status					
Spouse and Children	243	143 (58.8)	100 (41.2)	10.434	<0.05
Spouse	135	96 (71.1)	39 (28.9)		
Children	26	18 (69.2)	8 (30.8)		
Living Alone	16	6 (37.5)	10 (62.5)		
BMI					
Normal	150	91 (60.7)	59 (39.3)	3.16	>0.05
Overweight	197	130 (66.0)	67 (34.0)		
Obese	73	39 (54.0)	34 (46.0)		
Presence of chronic diseases					
None	139	127 (91.4)	12 (8.6)	73.352	<0.01
Present	281	136 (48.4)	145 (51.6)		
Family History					
None	293	205 (70.0)	88 (30.0)	22.344	<0.01
Present	127	58 (45.7)	69 (54.3)		
Stroke Location					
Non-Basal ganglion Region	247	175 (70.9)	72 (29.1)	17.357	<0.01
Basal ganglion Region	173	88 (50.9)	85 (49.1)		
Self-care Ability					
Fully Independent	103	95 (92.2)	8 (7.8)	96.674	<0.01
Mildly Dependent	235	150 (63.8)	85(36.2)		
Moderately to Severely Dependent	82	18 (22.0)	64(78.0)		
Number of Episodes					
First Episode	255	168 (65.9)	87(34.1)	3.945	>0.05
Second Episode	141	79 (56.0)	62 (44.0)		
Third Episode or More	24	16 (66.7)	8 (33.3)		
MRS Score					
1	211	173 (82.0)	38 (18.0)	93.052	<0.01
2	125	71 (56.8)	54 (43.2)		
3	82	19 (22.6)	65 (77.4)		
Perceived Social Support					
Low Support	29	7 (24.1)	22 (75.9)	130.659	<0.01
Moderate Support	186	79 (42.5)	107 (57.5)		
High Support	205	177 (86.3)	28 (13.7)		

### Correlation analysis

**Fig. 1:**
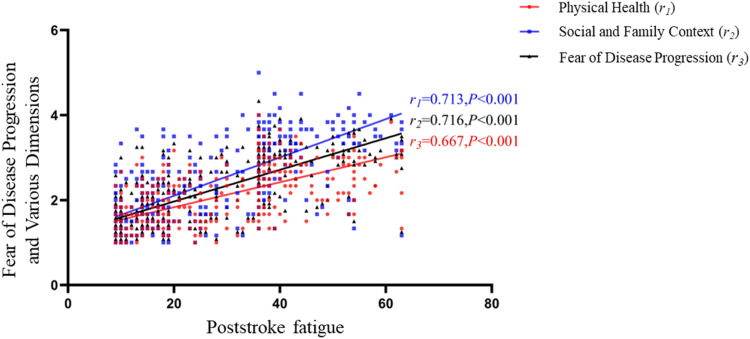
Correlation between PSF and fear of disease progression in patients with acute ischemic stroke.

The fear of disease progression score among stroke patients was (26.26 ± 9.42), with the physiological dimension score at (12.04 ± 4.00) and the social and familial dimension score at (14.22 ± 5.75). The fear of disease progression and scores for each dimension were positively correlated with PSF scores (all *p* < 0.01), as detailed in Fig. 1.

### Logistic regression analysis

The presence or absence of PSF was used as the dependent variable, and the total score for fear of disease progression, along with variables that showed statistical significance in univariate analysis, were used as independent variables in the unconditional logistic regression analysis. The results indicated that social support was a protective factor for PSF in patients with acute ischemic stroke while being female, having chronic diseases, stroke location in the basal ganglion, high MRS score, poor self-care ability, and fear of disease progression were identified as risk factors (*p* < 0.05), as shown in Table 3.

**Table 3:** Unconditional logistic regression analysis of PSF in patients with acute ischemic stroke (*n* = 420).

Factor	*b*	Sb	Wald χ^2^	p	OR	95 % CI
Constant term	−8.40	1.79	21.96	<0.01	‒	‒
Sex	1.51	0.42	12.78	<0.01	4.51	1.98∼10.29
Presence of chronic diseases	1.93	0.51	14.07	<0.01	6.86	2.51∼18.77
Stroke Location	0.97	0.41	5.50	<0.05	2.62	1.17∼5.88
Perceived Social Support	−1.56	0.37	17.91	<0.01	0.21	0.10∼0.43
MRS score	0.711	0.33	4.62	<0.05	2.03	1.06∼3.86
Self-care Ability	0.98	0.43	5.12	<0.05	2.66	1.11∼6.22

## Discussion

### Current status of PSF in patients with acute ischemic stroke

The results showed that the incidence of PSF among patients with acute ischemic stroke was 37.4 %, which is consistent with the findings of most studies.^9^ Items with higher scores included “My motivation is lower when I am fatigued” “Exercise brings on my fatigue” and “I am easily fatigued”. This indicates that PSF significantly impacts patients’ recovery adherence, energy, and physical strength, potentially leading to suboptimal recovery outcomes. Items with lower scores included “Fatigue is among my most disabling symptoms” and “Fatigue interferes with my work, family, or social life”. This may be due to the older age of the patients in this study, many of whom are retired or unemployed, so fatigue has less impact on their work. This suggests that healthcare providers should enhance their assessment of fatigue symptoms in patients and select appropriate intervention strategies based on the impact of fatigue to alleviate symptoms.

### Impact of demographic characteristics and disease conditions on PSF

The results indicate that female patients are more prone to PSF. Analyzing the underlying reasons, it is possible that there are differences in how men and women express or perceive fatigue symptoms, with females being more sensitive to their own discomfort. Chronic conditions such as diabetes and hypertension often lead to decreased compensatory capacity and reduced exercise endurance, which cannot meet the demands of patient rehabilitation training.^10^ Consequently, patients with comorbid chronic diseases are more likely to exhibit fatigue symptoms. The results of this study suggest that the risk of fatigue in patients with basal ganglion stroke is 2.624 times higher than in those with non-basal ganglion stroke. Most studies support that basal ganglion stroke is a risk factor for PSF, possibly related to the involvement of non-motor circuits in the basal ganglion.^11^ However, Maaijwee et al.^12^ did not find an association between the location of stroke and the occurrence of PSF, indicating ongoing controversy regarding the relationship between stroke location and PSF.

In this study, high MRS scores and poor self-care ability were identified as independent risk factors for PSF. This may be attributed to the fact that patients with poor neurological function and self-care abilities often experience negative emotions such as fear, anxiety, and depression due to restricted activities, which can indirectly contribute to the development of fatigue symptoms.^13^ High levels of social support are protective factors against PSF. This is likely because social support acts as a positive influence on the patient’s symptom experience. Patients with higher social support are more likely to benefit from substantial economic and emotional support, which effectively buffers the negative emotional experiences following a stroke.^14^ As nurses are the healthcare professionals most frequently interacting with patients, they should promptly assess the patient’s condition and psychological changes. Since fatigue is a self-perceived symptom, it is commonly present yet easily overlooked. It is recommended that clinical caregivers enhance the assessment of fatigue in patients with the aforementioned risk factors and inform physicians. This will assist in developing intervention strategies to reduce the negative impact of PSF on patients.

### Influence of fear of disease progression on the incidence of PSF

The results of the current study indicate a positive correlation between PSF scores and scores on the fear of disease progression, as well as the physiological health and social family dimensions. Fear of disease progression is a common psychological issue among stroke patients who may experience heightened concern or anxiety regarding their condition. Unlike anxiety or depression, fear is a more specific emotional response to chronic, debilitating, or life-threatening diseases. It is a valid psychological reaction; however, excessive fear can manifest as overly vigilant health monitoring or avoidance behaviors, severely impacting patients’ quality of life. Studies have indicated that fear of disease progression is a mediating factor in post-illness fatigue symptoms, potentially exacerbating fatigue by influencing patients’ confidence in their disease treatment or their perception of fatigue.^15^ In clinical practice, the management of PSF primarily involves both pharmacological treatments and non-pharmacological interventions, with the latter being the mainstay of care. Given the correlation between PSF and fear of disease progression, healthcare professionals, as the implementers of non-pharmacological interventions, may attempt to incorporate strategies for addressing fear into their care plans. Addressing fears could potentially reduce the incidence of PSF and mitigate its adverse effects.

### Study limitations and future directions

Our study has several limitations that warrant attention. First, the convenience sampling method used to recruit patients from a single tertiary hospital in Fujian Province may introduce selection bias, potentially limiting the generalizability of our findings to a broader population of acute ischemic stroke patients. Second, while validated, the reliance on self-reported measures such as the Fatigue Severity Scale (FSS) and the Fear of Progression Questionnaire (FoP-Q-SF) may be influenced by patients' subjective perceptions and emotional states, thereby affecting the objectivity of the results. Third, the cross-sectional nature of our study precludes establishing causal relationships between variables and assessing the longitudinal course of Post-Stroke Fatigue (PSF). Lastly, unexamined factors such as sleep quality, nutritional status, and genetic predispositions may also contribute to the development of PSF.

Considering these limitations, future research should focus on several key areas. Firstly, further exploration of the underlying biological mechanisms of PSF is necessary, particularly through integrating neurobiological, endocrinological, and immunological perspectives, which could identify novel biomarkers and therapeutic targets. Secondly, well-designed longitudinal studies are essential to track the natural history of PSF and evaluate the long-term impact of various interventions, incorporating a broader range of potential risk factors and outcome measures. Thirdly, multidisciplinary collaboration should be strengthened to develop comprehensive and personalized treatment strategies addressing the physical, psychological, and social aspects of PSF. Finally, international comparative studies could enhance our understanding of cultural and ethnic differences in PSF prevalence, risk factors, and management strategies, leading to more globally applicable guidelines.

## Ethics statement

The studies involving human participants were reviewed and approved by the Ethics Committee of The Affiliated Hospital of Putian University (n° 2023,074). The patients/participants provided their written informed consent to participate in this study. Written informed consent was obtained from the individual(s) for the publication of any potentially identifiable images or data included in this article.

## Funding

Funding This work was supported by 10.13039/501100009617Putian City Science and Technology Bureau in 2022 (No. 2022SM002).

## Declaration of competing interest

The authors declare no conflicts of interest.
